# Evaluation of physical parameters and spectral characterization of the quality of soaps containing by-products from the food industry

**DOI:** 10.1038/s41598-024-54794-3

**Published:** 2024-02-26

**Authors:** Patrycja Łusiak, Renata Różyło, Jacek Mazur, Paweł Sobczak, Arkadiusz Matwijczuk

**Affiliations:** 1 Department of Food Engineering and Machines, University of Life Sciences in Lublin, Akademicka 13, 20-950 Lublin, Poland; 2 Department of Biophysics, University of Life Sciences in Lublin, Akademicka 13, 20-950 Lublin, Poland; 3 ECOTECH-COMPLEX – Analytical and Programme Centre for Advanced Environmentally-Friendly Technologies, Maria Curie-Sklodowska University, Lublin, Poland

**Keywords:** Fourier-transform infrared spectroscopy, Soap, Pomace, Friction, Hardness, Cutting forces, Penetration work, Techniques and instrumentation, Plant sciences, Engineering, Materials science

## Abstract

The present study investigated several properties such as friction, hardness, penetration work, and cutting forces in soaps formulated with apple and carrot pomace at varying concentrations of 5%, 10%, and 15%. To gain insights into the molecular-level alterations within the formulated soap samples, they were spectroscopically analyzed using Fourier-transform infrared spectroscopy. The sliding friction analyses revealed that the investigated extrudate additives had no significant impact on the frictional forces of the soaps. However, notable differences were observed in the cutting force, hardness, and penetration work between the control and pomace-added samples. Excluding the control samples, no statistically significant distinctions were found between the cutting force, hardness, and work of penetration of soaps containing apple pomace and carrot pomace. Moreover, the quantity of pomace incorporated did not induce any significant variations in the results. The obtained samples were characterised at the molecular level using FTIR Fourier transform infrared spectroscopy. On the other hand, alterations in band intensities suggested improved molecular packing of the compounds within the samples due to the presence of the additives.

## Introduction

The increasing popularity of fruit-based products, including juices, purees, and smoothies, has led to a rise in the production waste known as pomace. Pomace refers to the remnants of fruits, vegetables, or seeds that result from the pressing of juices or oil. Effective disposal of the unused part of the raw material is a significant challenge for the food industry. Various studies highlight the potential usage of pomace across diverse industries^1^. In the food sector, these waste products are used as ingredients in fruit teas and as additives in confectionery and bakery products^2^.

In the cosmetic industry, extracts derived from pomace are ingredients in different cosmetic formulations, whereas, in the textile industry, pomace is utilized as an alternative raw material for the production of animal skin^3,4^.

Fruits and vegetables have a perfect blend of sugars, acids, and aromatic compounds developed and stored during their ripening process. These products are a rich source of vitamins, especially vitamins A and C, minerals, and bioactive compounds^5^.

For instance, apples and carrots are characterized by their high content of ursolic acid, ascorbic acid, malic acid, succinic acid, chlorogenic acid, and quinic acid. Beyond their nutritional benefits, these fruit organic acids can also be applied externally, directly to the skin^3,4^.

Organic acids are common exfoliating substances used in dermatology and cosmetology. They are used in the treatment of conditions such as acne, skin hyperpigmentation, and dead skin exfoliation procedures (commonly known as chemical peeling). During the exfoliation procedure, organic acids are applied to the skin surface in a controlled manner^6^. The depth of epidermal exfoliation depends on the concentration, the type of agent, and the duration of its contact with the skin^7^. Products with acid concentrations less than 4% work by weakening corneocytes adhesion, which facilitates the removal of the keratinized epidermis. Higher concentrations of acid in the products coupled with low pH induce epidermolysis by disrupting the desmosomal connections of the basal cell layer of the epidermis. To optimize the exfoliation potential of soaps, a synergistic approach can be used by combining both mechanical (by abrasion) and enzymatic (involving small amounts of acids in the pomace) exfoliation methods. This dual strategy if applied could enhance the efficacy of the exfoliation process^8,9^.

Although there is a lack of studies in the existing literature on the subject, there is a noticeable surge in the demand for soaps with natural additives. The ecological prospect of soap production using natural products presents a promising alternative to the synthetic chemicals used in traditionally formulated soaps^10,11^. Therefore, researchers have directed their focus toward the use of biomass waste for soap production.

To date, date syrup biomass waste has been used to produce soaps with enhanced antibacterial and antioxidant activity^12^.

Other studies have made use of agricultural wastes such as cocoa pod husks, palm bunch waste, sorghum husks, and peanut shells for the production of black soaps. Soaps formulated with these agro-wastes exhibited excellent solubility, texture, effective washing, and foaming abilities^13^. Other experiments utilized almond shells and orange peels and used cooking oil to craft soaps. Consumer acceptance studies confirmed the high potential of these additives in soap production^14^. Febriani et al.^15^ introduced an extract from waste oil palm leaves for the production of antibacterial soap, showcasing antimicrobial activity against *Escherichia coli* and *Staphylococcus aureus*. Furthermore, different aqueous extracts from household organic wastes including red beet, potato, papaya, lemon, and flowers were processed to formulate coconut oil-based soaps. These different soap formulations as the final product were chemical-free alkaline organic soaps with excellent foaming and cleaning properties^16^. These products enhanced the exploration of sustainable innovative soap formulations with different functional properties.

The objective of this research is to assess the feasibility of using by-products from the food industry, i.e., apple and carrot pomace, for cosmetic applications by introducing them as additives in soap production. In our study, additives in the form of carrot and apple pomace were selected to play the role of exfoliants due to their friction-inducing particles. The soaps formulated with these additives were subjected to quality assessment using specialized laboratory equipment.

To enhance the depth of analysis of the obtained samples, they were studied, assessed, and compared in terms of changes occurring at their molecular level with the help of Fourier-transform infrared spectroscopy (FTIR). FTIR is a noninvasive, fast, efficient, and, cost-effective research method. Its versatility allows us to assess the quality of materials of various origins along with their potential molecular changes and interactions in the samples influenced by the processing agents and their applied additives.

## Material and methods

### Material

The research utilized soap made in our in-house laboratory with the pomace generated using a prototype basket press designed by our team. The initial phase involved the preparation of a control soap mixture (base soap) without any additives, constituting 100% of raw material (Table 1). Subsequently, 5%, 10%, and 15% of carrot or apple pomace were incorporated into the whole mixture. Prior to molding, the soap mixture with the appropriate amount of pomace was thoroughly whipped up for a minute. The compositions of soap mixture were as follows: Apple pomace containing a dry matter content—80 g/100 g (d.m.), sugar content—1.2 g/100 g (d.m.), fiber content—6.7 g/100 g (d.m.), and protein content—0.66 g/100 g (d.m.). In comparison, the pomace of carrots contained a slightly higher proportion of dry matter—80.8 g/100 g (d.m.), approximately half the sugar content—0.68 g/100 g (d.m.), a small proportion of dietary fiber—0.4 g/100 g (d.m.), and a proportion of dietary protein—0.27 g/100 g (d.m.).

**Table 1:** The percentage composition of the base soap.

Ingredient	Amount (wt%)
Sunflower oil	20.2
Coconut oil	20.2
Olive oil	28.2
Beeswax	2.4
Distilled water	19.4
NaOH	9.6

To prepare the samples, all the ingredients specified in the recipe were measured. The solid fats (coconut oil and beeswax) were dissolved in a water bath, which means that they were placed in a separate vessel, which was placed in a vessel with heated waterThe fats in liquid form (sunflower oil and olive oil) were poured into a separate vessel. The alkali solution (NaOH in distilled water) was prepared and then cooled. Following the dissolution of solid fats, they were poured into a vessel with the liquid oils. Subsequently, NaOH and pomace were added and mixed thoroughly to achieve the desired consistency. The formulated soaps were poured into cylindrical silicon molds with a diameter of 26 mm (± 0.1 mm) and a height of 12 mm (± 1 mm) with a tare weight of 5 g (± 0.5 g). They were then cooled for 24 h. There were 114 soaps tested, which means that 18 soaps were tested for each sample.

### Measurement of the mechanical properties of soaps

The obtained soap samples with the addition of crushed pomace were subjected to comprehensive quality assessment by determining their mechanical parameters such as dynamic friction, measurement of the maximum cutting force, and resistance during sample penetration.

#### Measurement of the friction force

The evaluation of soap friction parameters was implemented using cylindrical soap samples with a diameter of 26 mm (± 0.1 mm), a height of 12 mm (± 1 mm), and a tare weight of 5 g (± 0.5 g).

Friction force measurements for each sample were conducted against a synthetic leather with the surface being moistened with distilled water (1 cm^3^ of water spread over the analyzed surface 10 s before the test) at a relative humidity of 40% ± 5%. The applied sample load was equal to 200 g. The measurements were performed on a modified friction bench as described in our earlier study^17^. The adjustment entailed varying the mounting and movement of the sample in order to better cooperate with the dimensions and form of the tested cylindrical samples while assuring parameter stability during measurements.

The measurements were calculated on the basis of a procedure developed by Stable Micro Systems: *'*Measuring bi-directional friction properties of materials using the Horizontal Friction System*'*. The measurement method was developed in compliance with the modified ASTM Standard Method D1894^18^. Dynamic friction and friction work during dynamic friction for a movement in a single direction were measured using this approach^17^.

The friction test was conducted with a test speed of 2.5 mm s^−1^. The measuring platform traveled a distance of 100 mm, and there was an initial displacement of 1 mm before initiating dynamic friction measurements. These measurements were conducted in 5 repetitions.

#### Measurement of maximum cutting force

**Figure 1:**
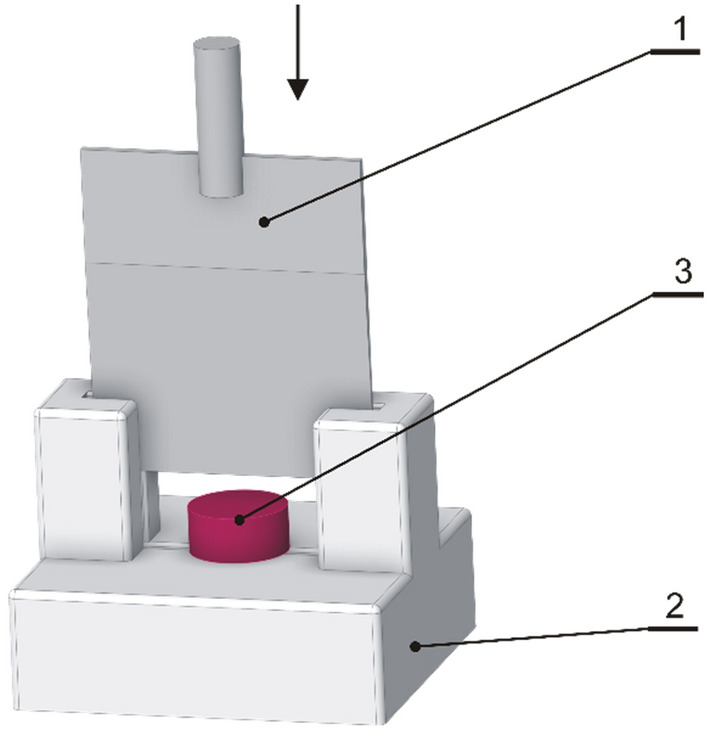
Arrangement of the sample during the measurement of the cutting force: 1-cutting blade, 2-base, 3-sample.

The cutting test was performed using a Stable Micro Systems TA.XT.Plus equipped with a 500N measuring head. Similar to the friction test, the cutting process involved cylindrical soap samples with a diameter of 26 mm (± 0.1 mm), a height of 12 mm (± 1 mm), and a tare weight of 5 g (± 0.5 g). A knife with a blade angle of 2.5° was employed for the cutting test, extending the entire length along the sample diameter (see Fig. 1). The cutting was executed with a knife and measuring the head speed of 5 mm s^−1^. Maximum cutting force values were recorded during the test and the results were read from the force–displacement diagram. All the measurements were conducted at ambient temperature (20 ± 1 °C), and the tests were carried out in 5 repetitions.

#### Measurement of resistance during sample penetration

**Figure 2:**
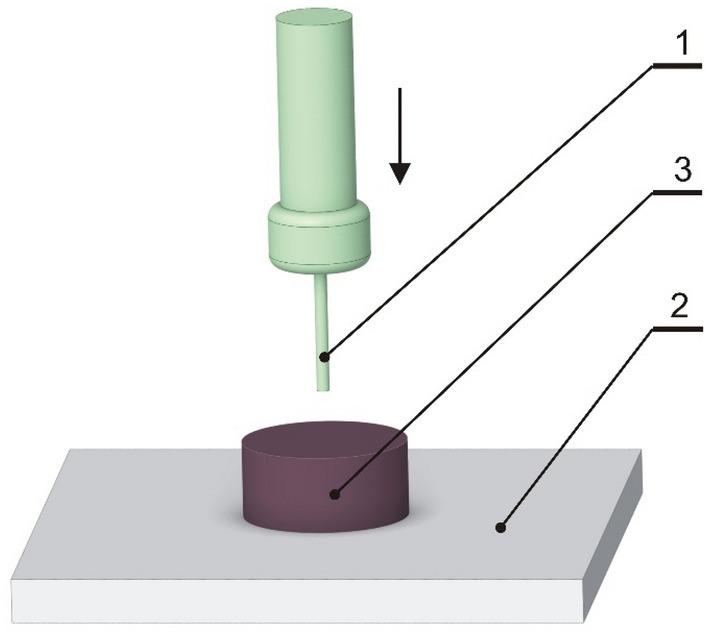
Arrangement of the sample during the hardness measurement: 1-penetrator, 2-base, 3-sample.

A soap sample, with the dimensions mentioned above, underwent resistance to measurement during penetration using a TA.XT.Plus device is equipped with a 500 N measuring head and a circular penetrator 2 mm diameter. The penetrator traveling speed was set to 1 mm s^−1^ to reach a depth of 5 mm. The hardness was determined as the maximum force recorded during the test and the results were read from the force–displacement diagram. All the measurements were conducted at ambient temperature (20 ± 1 °C), and the tests were carried out in 5 repetitions. The specimen arrangement is depicted in Fig. 2.

### Fourier-transform infrared spectroscopy (FTIR)

An IRSprit spectrometer by Shimatzu (Japan) was used for measuring FTIR spectra of the soap samples. An attenuated total reflection attachment in the form of a ZnSe crystal. Each spectrum was measured in the spectral range from 450 to 3800 cm^−1^, and the instrument resolution was set to 2 cm^−1^. Spectral analysis and preparation of the spectra for publication was performed using Grams AI software by Thermo Galactic Industries (USA), as well as Origin Pro 2021 by OriginLab Corporation (USA). Grams AI software was also used in the preliminary analysis of the spectra to prevent interpretative mistakes. This entailed a baseline cutoff and realignment of the spectra at a single reference level for ease of interpretation. The background was measured and the spectra averaged using software provided by the manufacturer. The obtained spectra did not require additional processing that could potentially affect the interpretation, e.g. smoothing. Origin Pro 2021 software was used to present the infrared spectra registered in this study.

### Statistical analysis

The statistical analysis of study results was carried out using Statistica 13 by StatSoft, employing a two-factor analysis of variance considering the account share and type of pomace. The normality of distribution was tested using the Shapiro–Wilk test and the group homogeneity was determined using the Tukey test at a significance level of *α* = 0.05.

## Results and discussion

The conducted analyses aimed at assessing the quality of using fruit pomace in sodium soaps.

### The results of sliding friction examination of the tested soaps

**Figure 3:**
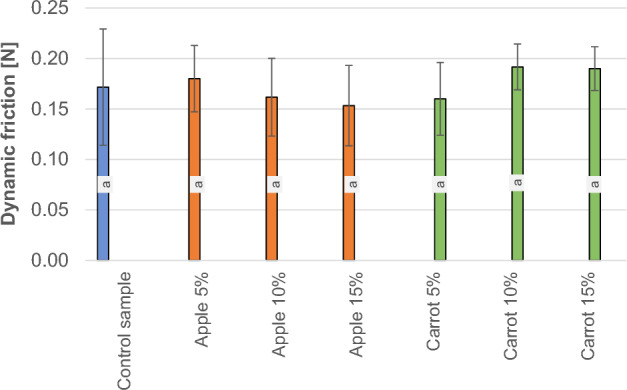
Results of the dynamic friction measurement of soaps with various fruit pomace additives, (**a**, **b**) mean values marked with different letters are significantly (*α* = 0.05) different.

Figure 3 summarises the results obtained from the sliding friction force measurements for the individual samples of apple and carrot pomace soaps within the tested range of admixture between 5 and 15%.

The findings from the sliding friction (dynamic friction) examination showed no statistically significant differences between the control soap sample and the formulated soap blends with varying amounts of apple and carrot pomace. The observed low friction force of the soap samples can be due to the pre-wetted surface and the specificity of the produced samples. Therefore, it can be assumed that the addition of pomace did not exert a discernible impact on the friction force.

Yet, it is noteworthy that the type and condition of the friction surfaces significantly influence the maximum static frictional force^19^. Although there is a lack of specific studies on the frictional force of different types of soap, research on biological materials, such as wheat, indicates that static friction coefficients of wheat grains against galvanized sheet metal, plywood, and plexiglass varied significantly for different contact surfaces^20^. Kaliniewicz et al.,^21^ determined the coefficient of static friction against the steel surface for different grains and seeds and noted that the coefficient of static friction was correlated mainly with seed thickness. Meanwhile, Zhang et al.^22^ conducted a parametric study to understand the impact of dimensionless coating thickness and the material properties of the coating and the surface on the static friction coefficient.

Information on the impact of the type of construction material and the working surface roughness on the friction of biological material particles, mainly concerns the materials used for the construction of certain equipment, its working elements, and storage facilities. Research findings in this domain mainly relate to materials such as steel, wood^6,20^, rubber^20^, PVC, and aluminum. Crucial factors that have a decisive impact on the friction of plant materials include the normal pressure, the type of construction material, the surface roughness of the friction materials, the species (variety) of plant material, the moisture content of the plant material, and the orientation of the biological material tested to the direction of their movement^23^. In the case of these tests, the influence of changing pressure on friction parameters was not taken into account. In our study, we observed that the values of friction force were not statistically different between soaps with the addition of food pomace and natural soaps, i.e., without the admixture thereof. That is, the additive did not affect the quality of the soap measured using friction parameters. If we want to achieve a peeling effect, the friction parameters can be higher, but in the case of traditional soap, it is important that it be pleasant to the touch and easy to apply to the skin during use^24^.

### Cutting force

**Figure 4:**
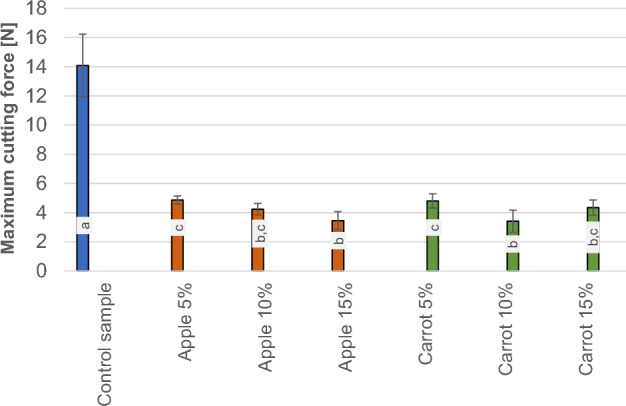
Results of the cutting force measurement of individual samples.

Figure 4 shows the results of the measurements of cutting force values for the individual samples.

It can be observed from the graph that the control sample exhibits the highest cutting force with a value of 14.1 N. In contrast, the cutting forces recorded for the soap with apple pomace were 4.9 N, 4.2 N, and 3.5 N, whereas the cutting forces for the soaps with the carrot pomace additive were 4.8 N, 3.4 N, and 4.4 N, respectively. Statistical analysis demonstrated that there were no significant differences in the cutting force between the samples with apple and carrot pomace admixture. However, statistically significant differences occurred between the control sample and the soaps with pomace addition. Remarkably, a small addition of pomace (5%) resulted in a substantial reduction in cutting force values. Yet, an increase in the amount of fruit pomace admixture from 5 to 15% did not result in a further reduction in the cutting force values. According to different literature on the subject, up until now, no cutting forces tests have been carried out for soaps with various additives. It should be noted that the cutting force of any material depends on its properties such as hardness, plasticity, or brittleness^25^. Food pomace derived from apples and carrots is a biological material with a much lower hardness than the control soap, because of which their addition had such a significant effect on the cutting force. In terms of soap quality, a lower cutting force will be beneficial in the soap production process, and less energy will be needed for its production. In addition, as other authors have noted, hard soap, unlike soft soap, is difficult to dissolve in water^26^.

### Resistance to penetration

**Figure 5:**
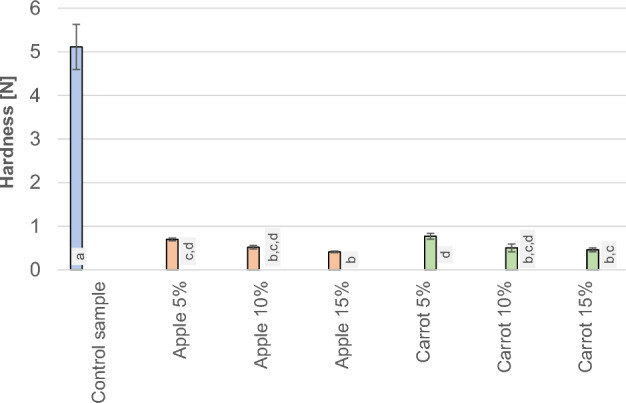
Results of the measurements of penetration resistance values for individual samples.

Figure 5 shows the changes in the penetration resistance force for the test samples.

A similar pattern to the cutting force values was obtained in the measurement of penetration resistance (see Fig. 5). The control sample exhibited the highest resistance value of 5.1 N, whereas the others had values below 1 N. Penetration resistance, akin to cutting force, is a parameter that depends on the hardness, plasticity, or brittleness of the material. Therefore, there was a substantial decrease in its value upon the addition of carrot or apple pomace to the soap. In the case of soaps, penetration resistance is a physical parameter that affects its quality, i.e. the soap abrasion process. Probably, it should be easier for the soap to dissolve and saponify after contact with the skin.

### FTIR spectroscopy

**Figure 6:**
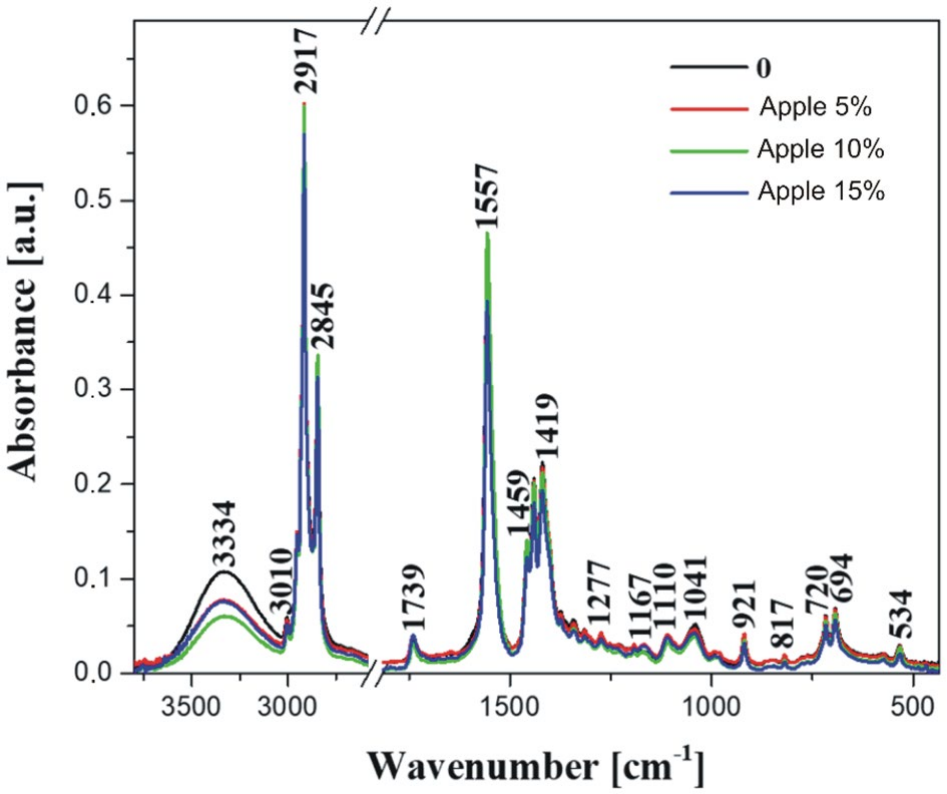
FTIR infrared spectra of the tested soap samples, made in the spectral range: 450 cm^−1^ to 3750 cm^−1^: 0—control sample and samples with 5%, 10%, and 15% of apple pomace admixture, respectively.

**Figure 7:**
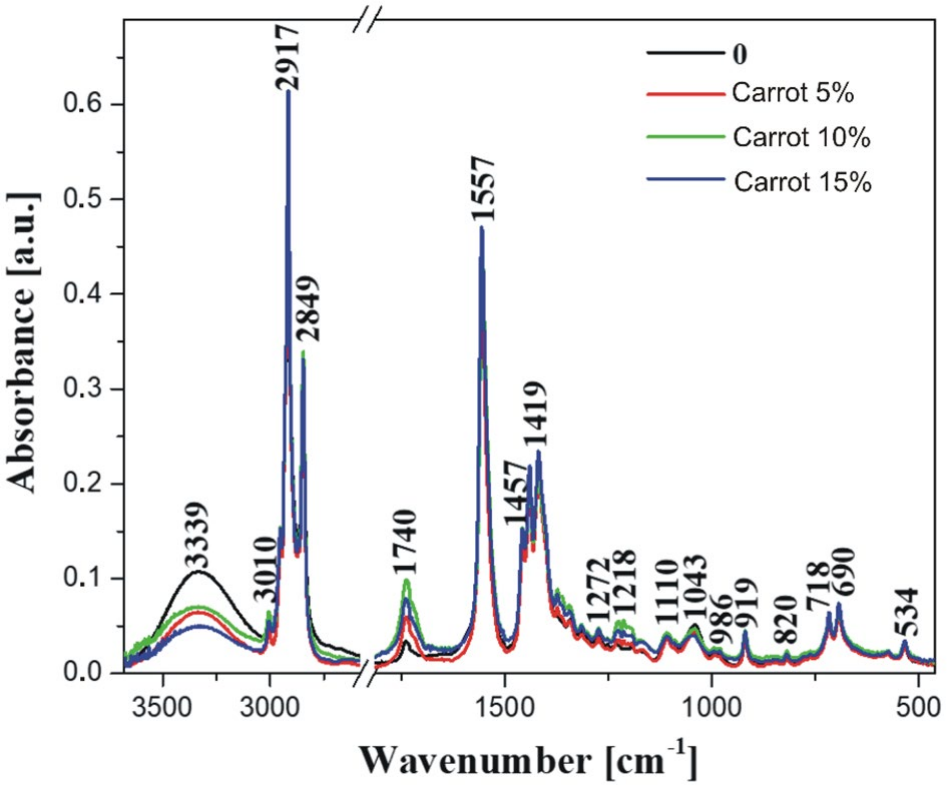
FTIR infrared spectra of the tested soap samples, made in the spectral range: 450 cm^−1^ to 3750 cm^−1^: 0—control sample and samples with 5%, 10%, and 15% of carrot pomace admixture, respectively.

The infrared FTIR spectra obtained for the formulated samples of soaps with apple pomace (see Fig. 6) and carrot pomace (see Fig. 7) are presented in corresponding figures. Additionally, Table 2 provides a comprehensive overview of the most important and characteristic bands with the corresponding functional groups assigned to them^27–35^. The presented spectra of modified soaps distinctly show the spectral backbone characteristic of these substances. Despite the identical principal structure of the soap-forming molecule across all the samples, differences are mainly due to the added pomace, and the resulting molecular interaction and arrangement in the presence of the said admixture. The available literature on this subject attempts to accurately assign the vibrations of specific functional groups to bands in similar materials, obtained through various methods^27,29^. However, it is quite difficult to interpret and assign bands to a particular functional group due to the variety of additives used and the specific modifications to a given product.

**Table 2:** The location of the maxima of the FTIR absorption bands, with the assignment of particular vibrations to the respective samples: control and with 5%, 10%, and 15% of apple and carrot pomace admixture^27–35^.

FTIR		Type and origin of vibrations
Positioning of band (cm^−1^)		
Apple	Carrot	
3330	3332	ν_st_(O–H) and –OH in H_2_O
3005	3009	ν_s+as_(C–H) in CH_2_ and CH_3_ group in fatty acids
2955	2953	
2911	2914	
2845	2847	
1740	1739	ν (C = O) and ν (C = O)^**…**^OH
1714	1720	
1554	1556	ν_a_ (COO)
1459	1459	δ (CH_2_) + ν_s_ (COO)
1437	1443	
1419	1417	
1371	1375	δ (CH_2_) + ν (C–C)
1343	1343	
1316	1315	
1272	1274	
1225	1231	
1199	1194	
1166	1163	
1112	1107	ν (C–C) + δ (C–C–C)
1043	1043	δ (CH_2_)
992	993	
918	918	ν (C–C) carboxyl
816	816	δ (CH_3_) + ν (C–C)
762	762	ν (C–C)
716	717	δ (COO)
691	694	
573	572	
534	531	

Ν, stretching vibrations; δ, deformation vibrations; s, symmetric; as, asymmetric; st, strong.

We began the analysis by characterizing important bands in the obtained spectra of the samples modified with the additive of apple and carrot pomace. On the long-wave side, a highly characteristic band was observed with a maximum at ~ 3334 cm^−1^, originating from the stretching of –OH hydroxyl groups. These –OH hydroxyl groups were present in abundance in the molecules of our studied product samples^27^. The enhancement of the spectra by the vibrations of free water molecules and the intensity may result in a slight shift to the short-wave side depending on the affinity of the molecules to form hydrogen bonds with the additives used. Furthermore, we observed a characteristic –C–H stretching vibrations (likely from different cis- and trans- transformations) with the maxima peaks at ~ 3010 cm^−1^, 2955 cm^−1^, 2917 cm^−1^, and 2845 cm^−1^ (Figs. 6 and 7). These vibrations stem from the groups in the long hydrocarbon chain molecules in the fat fraction of the studied samples^27,29,32^. In such samples, vibrations are usually prominent due to the large number of these groups in the hydrophobic part of the molecules^29^. They originate from the –C–H stretching vibrations in the –CH_3_ and –CH_2_ groups belonging to the aliphatic groups found in both symmetric and asymmetric hydrocarbon chains. Another very interesting sharp band was characterized with relative intensity at ~ 1739 cm^−1^, representing a stretching vibration of the –C=O carbonyl group^27,28,31,32^ in the hydrophilic part of the studied soap molecules. One can observe a broadening of this band on the shorter wavenumber side with the maximum at ~ 1715 cm^−1^, potentially originating from hydrogen-bonded carbonyl groups, probably in the C = O…H–O– configuration^35^ or due to the interaction of the polar head of the molecule with other groups. The visibility of this band indicates that there is a significant number of such structures, confirming the dense packing of molecules in the soap samples obtained, especially in the ones where the additives were used. This fact can indirectly indicate a positive quality of the obtained product. Further, a sharp and intense band was observed around 1557 cm^−1^ likely arising from –C=C stretching vibrations in the cis-transformation^29^. However, these vibrations can also be attributed to asymmetric stretching vibrations of the –COO grouping^27,28,31^. The range of vibrations from 1465 to 1410 cm^−1^ is notable for the first two intense and sharp bands, which belong to the deformation vibrations in the –CH_2_ group, while the band with the maximum at 1419 cm^−1^ is a symmetric stretching vibration in the –COO group^27^, forming the hydrophilic “head” structure of the obtained soap molecule. Similarly, a spectral range of vibrations was observed from about 1410 cm^−1^ to 1150 cm^−1^, belonging mainly to –CH_2_ deformation vibrations present in a significant amount in soap molecules^27^, primarily in the hydrocarbon chain of their hydrophobic part. Two bands with peaks 1110 cm^−1^ and 1041 cm^−1^ were also highlighted with the former as a superposition of –C–C stretching vibrations with –C–C–C deformation vibrations, whereas the latter is most likely another harmonic of deformation vibrations in the –CH_2_ grouping. The region below 1000 cm^−1^ in the final characterization of the measured spectra was responsible for the vibrations in the linkage structures of the molecules or the presence of overtones of other previously observed vibrations. The characteristic vibration with the maximum at 921 cm^−1^ is attributed to a –C–C stretching vibration in the carboxyl group. At the same time, a faint intense vibration at 817 cm^−1^ combines deformation vibration in the –CH_3_ group with stretching vibrations in the –C–C group. Vibrations in the area below 730 cm^−1^, particularly at two sharp peaks 720 cm^−1^ and 694 cm^−1^, and 534 cm^−1^ are linked to deformation vibrations in the –COO system, resembling overtones of previously observed vibrations^27^. However, the precise definition of this phenomenon remains indeterminate in the existing literature.

By way of a preliminary summary of the results obtained with the use of FTIR infrared spectroscopy, one should note the differences in the character of the bands registered for the samples with the addition of both carrot and apple pomace associated primarily with changes in the intensity of particular bands. No significant changes in terms of spectral shifts of the bands were observed. This may indicate absence of a negative impact on the primary structure of the obtained soap samples. The slight shifts recorded for the samples with the addition of apple or carrot pomace were most likely associated with the various sugar fractions present in the given pomace additive. The addition of the carrot or apple pomace was observed to increase the intensity of certain bands, mainly those with the maxima at ~ 2917, 2845, 1557, respectively, or within the range from 1465 to 1400 cm^−1^. At the same time, we observed a decrease in the intensity of vibrations below 1100 cm^−1^. The respective bands yielded interesting observations that correspond to very interesting effects. The increase in the intensity of the aforementioned bands was associated with an increase in the sugar fraction of the analyzed samples, as confirmed by the elevated intensity of the specific vibrations mentioned above. This was directly related to the amount of the additive used. The observed effects fully support the chosen research direction, which we intend to further pursue in the future by incorporating additional, more advanced analytical techniques, such as microstructural analyses. The results complement and align with those obtained from the physical analyses described above.

## Conclusions

The findings from the research on the physical properties of the soaps showed that the incorporation of apple and carrot pomace did not lead to significant changes in the dynamic friction coefficient. In the case of apple additives, the sliding friction force (0.18 N, 0.16 N, and 0.15 N) decreased compared to the control sample (0.17 N). On the other hand, in the case of carrot additives, the value increased with the number of added carrots (0.16 N, 0.19 N, and 0.19 N). Statistical analysis showed no significant differences between the individual parameters, while the cutting force and penetration resistance of the soaps decreased significantly with just a 5% admixture of both apple and carrot pomace. The highest cutting force was characterized in the control sample with 14.1 N. On the other hand, the cutting forces of soap with the addition of apple pomace were 4.9 N, 4.2 N, and 3.5 N and soap with the addition of carrot pomace were 4.8 N, 3.4N, and 4.4N. Statistical analysis showed no significant differences in cutting force between the samples with the addition of pomace but significant differences occurred between the control sample and soaps with the addition of pomace. A higher proportion of pomace in the range between 5 and 15% did not cause further substantial changes in the cutting force and penetration resistance of the soaps. (The highest penetration work occurred for the control sample which was measured to 18 mJ). Obtaining lower values of cutting force, hardness and penetration resistance, may be beneficial because such soap will be more pleasant to the touch and dissolve more easily in water after contact with the skin.The sliding friction tests for understanding the physical and mechanical properties of added apple and carrot pomace showed no significant differences in the amount of the introduced admixture. However, further analysis using FTIR infrared spectroscopy confirmed that the most substantial changes in the obtained samples occurred with the increase of the pomace admixture in the soaps. The changes in the spectra were visible in terms of the intensity of the bands and indicated a lack of negative effects on the internal structure of the formulated samples. It also confirmed the quality as well as the benefits of the additives used.

In summary, the research emphasized the impact of apple and carrot pomace additives on soap quality properties, encompassing mechanical parameters and spectroscopic characteristics. FTIR spectroscopy measurements indicated changes in intensity within relevant bands, potentially serving as a rapid spectroscopic marker for the additives used, even in trace amounts, unlike other more costly measurement methods.

## Data Availability

The datasets used and/or analyzed during the current study available from the corresponding author on reasonable request.
