# Author Correction: Evaluation of physical parameters and spectral characterization of the quality of soaps containing by-products from the food industry

**DOI:** 10.1038/s41598-024-64152-y

**Published:** 2024-06-11

**Authors:** Patrycja Łusiak, Renata Różyło, Jacek Mazur, Paweł Sobczak, Arkadiusz Matwijczuk

**Affiliations:** 1 Department of Food Engineering and Machines, University of Life Sciences in Lublin, Akademicka 13, 20‑950 Lublin, Poland; 2 Department of Biophysics, University of Life Sciences in Lublin, Akademicka 13, 20‑950 Lublin, Poland; 3 ECOTECH‑COMPLEX – Analytical and Programme Centre for Advanced Environmentally‑Friendly Technologies, Maria Curie-Sklodowska University, Lublin, Poland

Correction to: *Scientific Reports* 10.1038/s41598-024-54794-3, published online 26 February 2024

The Funding section in the original version of this Article was omitted. The Funding section now reads:

“Publication co-financed by the state budget under the program of the Ministry of Education and Science (Republic of Poland) under the name Excellent Science - Support for Scientific Conferences entitled “XXIII Polish Nationwide Scientific Conference “PROGRESS IN PRODUCTION ENGINEERING” 2023” project number DNK/SP/546290/2022 amount of funding 162650,00 PLN total value of the project 238 650,00 PLN (Poland).”

The original Article has been corrected.

